# Drosophila *brca2* Is Required for Mitotic and Meiotic DNA Repair and Efficient Activation of the Meiotic Recombination Checkpoint

**DOI:** 10.1371/journal.pgen.0040031

**Published:** 2008-02-08

**Authors:** Martha Klovstad, Uri Abdu, Trudi Schüpbach

**Affiliations:** Howard Hughes Medical Institute, Department of Molecular Biology, Princeton University, Princeton, New Jersey, United States of America; Stowers Institute for Medical Research, United States of America

## Abstract

Heterozygous mutations in the tumor suppressor *BRCA2* confer a high risk of breast and other cancers in humans. BRCA2 maintains genome stability in part through the regulation of Rad51-dependent homologous recombination. Much about its precise function in the DNA damage responses is, however, not yet known. We have made null mutations in the Drosophila homolog of *BRCA2* and measured the levels of homologous recombination, non-homologous end-joining, and single-strand annealing in the pre-meiotic germline of Drosophila males. We show that repair by homologous recombination is dramatically decreased in Drosophila *brca2* mutants. Instead, large flanking deletions are formed, and repair by the non-conservative single-strand annealing pathway predominates. We further show that during meiosis, Drosophila Brca2 has a dual role in the repair of meiotic double-stranded breaks and the efficient activation of the meiotic recombination checkpoint. The eggshell patterning defects that result from activation of the meiotic recombination checkpoint in other meiotic DNA repair mutants can be strongly suppressed by mutations in *brca2*. In addition, Brca2 co-immunoprecipitates with the checkpoint protein Rad9, suggesting a direct role for Brca2 in the transduction of the meiotic recombination checkpoint signal.

## Author Summary

Breast cancer can arise due to inherited mutations in a few well-defined breast cancer susceptibility genes. *BRCA2* is one of two known human genes in which common mutations are associated with high breast cancer risk. A known function of BRCA2 is the repair of damaged DNA using the homologous recombination repair pathway. In the absence of BRCA2, damaged DNA is repaired by error-prone repair. However, as many other genes required for homologous recombination are not known breast cancer susceptibility genes, BRCA2 likely has additional roles in tumor suppression. In this study, we created fruit flies mutant for the homolog of *BRCA2*. We show that, like in humans, fruit fly Brca2 is required for DNA repair by homologous recombination. Additionally, we use this new animal model to discover a previously unknown role for Brca2 in the production of a signal that tells cells that damaged DNA is present during meiosis. This “checkpoint” signal may allow the cells to coordinate a protective response after DNA damage.

## Introduction

The genomic stability of eukaryotic cells is constantly challenged by exogenous and endogenous stresses that can lead to the loss or alteration of genetic material. Genomic stability is maintained through robust DNA repair and checkpoint pathways that are tightly coordinated with each other and the developmental cell cycle progression of the organism. For example, mutations in meiotic DNA repair enzymes in Drosophila cause defects in the cell cycle and developmental progression of the egg due to a failure to repair meiotic recombination intermediates [1]. The tumor suppressor and breast cancer susceptibility gene, *BRCA2*, has been implicated in playing a central role in maintaining genomic stability, but the extent to which BRCA2 is involved the coordination of DNA repair, checkpoints, and developmental progression remains to be determined.

Murine cells depleted for BRCA2 spontaneously accumulate broken chromosomes and chromatids, triradial and quadriradial structures, and gross chromosomal rearrangements [2,3]. A key function of BRCA2 is the regulation of the Rad51 recombinase during DNA repair by homologous recombination (HR). During HR, Rad51 assembles into a nucleoprotein filament with single-stranded DNA at the site of a double-stranded break (DSB) in order to initiate strand invasion of the homologous chromosome [4]. Recent structural studies have illuminated how BRCA2 regulates Rad51 [5–7]. BRCA2 contains two regions that mediate binding to Rad51: a stretch of 8 repeated short motifs termed the BRC repeats and a C-terminal region termed TR2. The BRC repeats bind the Rad51 oligermerization domain to disrupt Rad51 self-oligermerization. BRCA2 then catalyzes the formation of the nucleoprotein filament at the single-stranded/double-stranded DNA junction flanking a DSB. This filament is stabilized in a cell-cycle–dependent manner by the TR2 domain of BRCA2. The role of BRCA2 in homologous recombination is likely critical for its role as a tumor suppressor, but BRCA2 is a large protein with many binding partners and it is likely to play multiple roles in safeguarding genomic stability.

Several requirements for BRCA2 outside of homologous recombination have been suggested by protein interaction and cell culture studies, but these functions are far less understood. Most notably, BRCA2 has been implicated in two S-phase checkpoints: the intra-S phase checkpoint and the replication checkpoint. During the intra-S phase checkpoint irradiation-induced lesions outside of the replication fork cause partial depression of replication [8]. The replication checkpoint stabilizes replication forks and decreases replication levels in response to lesions at the replication fork. Requirements for BRCA2 in replication fork stabilization after hydroxyurea treatment and in suppressing radioresistant replication have also been described [9,10]. The G1/S, S/M, and G2/M checkpoints have been found to be largely intact in *BRCA2* mutants. However, these studies have used hypomorphic mutations that preserve half or more of the N-terminal region of BRCA2 due to proliferative defects of *BRCA2* null mutants [3,10]. As modest to severe increases in breast cancer susceptibility have been associated with mutations in other checkpoint genes [11], further elucidation of the mechanism of the role of BRCA2 in checkpoints is needed.

Drosophila has emerged as a useful model for studying DNA repair and checkpoint control of genome stability. The DNA repair and checkpoint pathways are remarkably well conserved between flies and higher organisms [12]. Notably, though the function of many of these genes is well conserved, null mutants in Drosophila are sometimes viable in cases in which null mutations in higher organisms result in lethality and complicate mammalian developmental studies [13,14]. We made null mutations in the Drosophila homolog of *BRCA2 (CG30169)*, and show that unlike in mammals, Drosophila *brca2* null mutants are viable. We present detailed descriptions of DSB repair pathway balance and irradiation-induced checkpoint function in animals genetically null for *brca2*. *CG30169* represents a functional *BRCA2* homolog required for DSB repair in mitotic and meiotic tissues. Additionally, we uncover a novel role for *brca2* in the meiotic recombination checkpoint. Finally we show that Brca2 co-immunoprecipitates with the checkpoint protein Rad9, suggesting a mechanism for the role of Brca2 in checkpoint control.

## Results

### Null Mutations in the Predicted Drosophila *BRCA2* Homolog, *CG30169*, Are Viable

Poor sequence conservation of BRCA2 homologs outside of the BRC repeats and DNA binding domains initially hampered the identification and study of BRCA2 in lower model organisms. However, by using a sequence motif that describes the BRC repeats, Lo et al. (2003) identified putative homologs across many species [15], including some that have since been verified to be functional homologs [16,17]. One gene in Drosophila, *CG30169*, was predicted to encode BRC repeats. *CG30169* encodes a 109 kDa protein containing 3 putative BRC repeats and 4 Nuclear Localization Sequences. Notably, while Lo et al. (2003) predicted DNA binding domains in vertebrate, plant, and some lower eukaryotes, they did not find evidence of a DNA binding domain in *CG30169* by sequence analysis. Therefore it was not self-evident that *CG30169* encodes a functional BRCA2 homolog, since the expression of isolated repeats is known to dominantly inhibit Rad51-dependent DSB repair, but fusions of a BRC repeat to Replication Protein A restore DNA binding and are consequently able to rescue the repair defects of *BRCA2* mutants [18–20].

In order to determine if *CG30169* encodes a functional BRCA2 homolog and examine its role in the DNA damage responses, we made null mutations in *CG30169*. *brca2* ^56E^ was created by imprecise excision of the P element *P(SUPor-P}CG30169^KG03961^* which is inserted in the 5'UTR of *CG30169*. *brca2* ^56E^ removes the majority of the coding sequence of *CG30169* and a portion of the 5'UTR of the neighboring gene *CG4612*. *brca2* ^KO^ was created by ends-out homologous recombination [21], and the resulting chromosome is completely deleted for *CG30169* but contains fully intact neighboring genes. We found that homozygous *brca2* ^KO^, *brca2* ^56E^, and transheterozygous mutant combinations are viable but recessive female sterile.

### The Drosophila *BRCA2* Homolog Is Required for Homologous Recombination Repair

The best characterized role of BRCA2 is in the regulation of the recombinase Rad51, which has been shown to be required for DNA repair in Drosophila [14]. In order to determine if *CG30169* represented a functional *BRCA2* homolog we first tested for a role in somatic DNA repair by assaying sensitivities to the mutagens, methyl methanesulfonate (MMS) and x-rays. X-ray irradiation (IR) results in a range of DNA damage including a large number of DSBs throughout the cell cycle, while MMS leads to replication fork collapse and DSB formation specifically during S phase. We found that similar to *rad51/spnA* and *rad54/okra* mutants ([14] and Tables 1 and 2), *brca2* mutants are highly sensitive to both MMS and IR. Flies heterozygous for either *brca2* allele were crossed together, the larval progeny were exposed to mutagen, and the percent of homozygous mutant flies that survive to adulthood among all surviving adults was determined. *brca2* mutants showed complete to near-complete lethality after a 1250 Rad IR exposure and 3–13-fold decrease in survivorship after exposure to 500 Rads of irradiation (Table 1). Exposure to 0.08% MMS resulted in a 5–24-fold decrease in survivorship of *brca2* mutants (Table 2). Flies doubly mutant for *okra* and *brca2* showed mutagen sensitivities comparable to the single mutants alone, suggesting the two genes act in the same pathway. The similar mutagen sensitivities between *CG30169*, *okra*, and *spnA* mutants suggest that *CG30169* may be a functional *BRCA2* homolog involved in DSB repair.

**Table 1:**
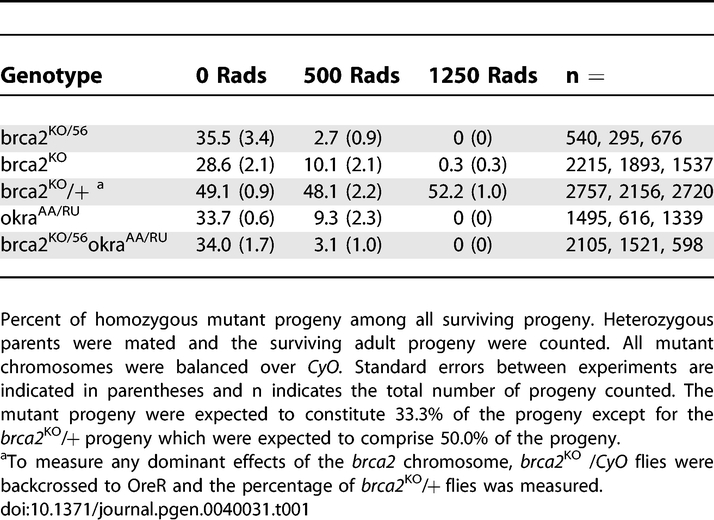
Irradiation Sensitivity of *brca2* Mutants

**Table 2:**
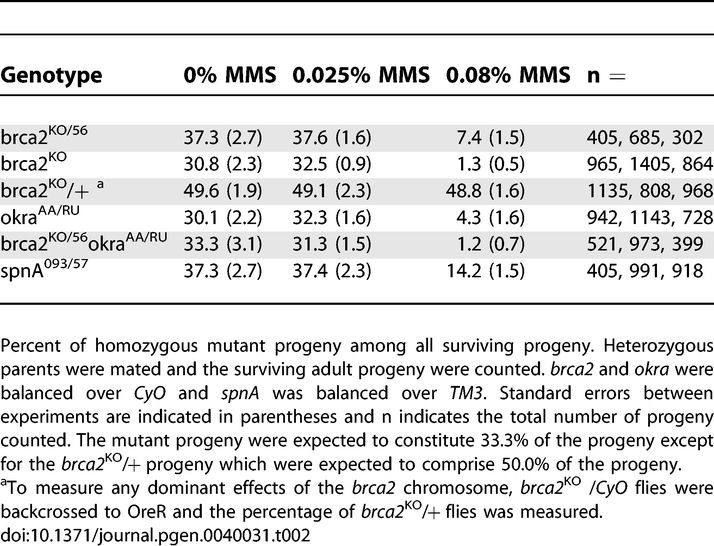
Methyl Methanesulfonate Sensitivity of *brca2* Mutants

Several pathways are responsible for the repair of DSBs and the choice between these pathways is a highly regulated process influenced by factors such as the nature of the break or the phase of the cell cycle [22,23]. Three major pathways known to repair DSBs are: non-homologous end-joining (NHEJ), homologous recombination (HR), and single-strand annealing (SSA) [4]. In NHEJ, the DNA ends flanking the break are annealed, resulting in the insertion or deletion of several nucleotides at the site of the break. During SSA repair stretches of repetitive DNA flanking the DSB are annealed. Due to the highly repetitive nature of eukaryotic chromosomes this pathway is likely available to some, but not all, naturally occurring DSBs. HR uses homologous sequences on the homologous chromosome or sister chromatid as a template for repair. If the sister chromatid is used, HR is non-mutagenic. However, if the homologous chromosome is the template, loss of heterozygosity can occur. BRCA2 in other species has been shown to be required for Rad51-dependent repair by homologous recombination and other roles in DNA repair have been suggested [16,17,24]. In C. elegans there is in vitro and indirect in vivo evidence for a role for the *C. elegans* BRCA2 homolog in SSA [16,25], though in mammalian cell culture studies with hypomorphic mutations, an increase in SSA was observed [26–29].

**Figure 1:**
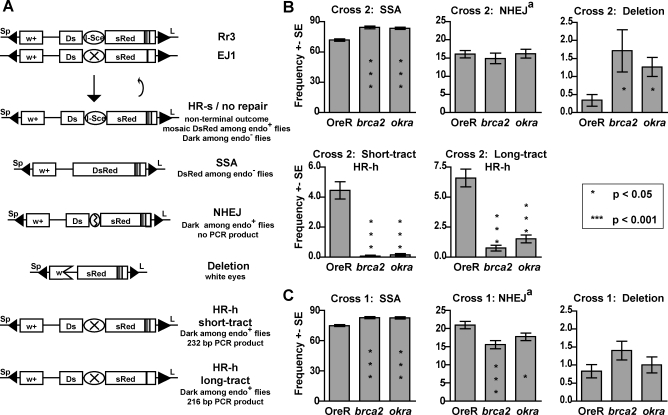
*brca2* Mutations Dramatically Decrease HR-h Repair, Increase SSA Repair, and Result in Increased Deletion Formation (A) The Rr3 assay measures the relative repair pathway usage for repair of I-SceI endonuclease-cut breaks in the pre-meiotic germline of Drosophila males. The Rr3 transgene contains an I-SceI site flanked by two partial copies of *dsRed*. One *dsRed* copy contains a 147 bp duplication. Repair by SSA reconstitutes *dsRed* and results in red fluorescent progeny. Repair by NHEJ modifies the I-SceI site and results in progeny that are dark regardless of the presence of the endonuclease. The EJ1 transgene is a variant of Rr3 which has a non-functional I-SceI site and a 16 bp deletion 156 bp from the I-SceI site. Progeny resulting from repair by HR-h are also dark even when the endonuclease is present and can be distinguished from NHEJ by PCR. PCR analysis also distinguishes between short and long tract HR-h; long-tract HR-h results in the inclusion of the deletion on EJ1 and a smaller product. Deletions are measured by the loss of the *w* ^+^ marker. (B) In Cross 2, the EJ1 chromosome was present, and repair by HR-h could occur. Error bars represent the standard errors between individual males; *P*-values were calculated using a permutation test described in [31]. Number of males/total Rr3-carrying progeny counted for OreR, *brca2*, and *okra*, respectively: 72/3861, 72/3163, and 78/3486. (C) In Cross 1, the EJ1 chromosome was not present, and repair by HR-h was not available. Number of males/total Rr3-carrying progeny counted for OreR, *brca2*, and *okra*, respectively: 66/7103, 67/6054, and 65/5546. ^a^The deletion class was not isolated prior to sorting and therefore represents a small portion of the NHEJ class.

We used the Repair Reporter 3 (Rr3) assay [30] to simultaneously measure the relative levels of HR, NHEJ, and SSA repair in the pre-meiotic germline of individual males (Figure 1). The Rr3 assay monitors the repair of a DSB at an I-SceI endonuclease site flanked by partial copies of the reporter *dsRed.* There is a 147 base pair (bp) tandem duplication of *dsRed* flanking the break. I-SceI is ubiquitously expressed at the onset of zygotic transcription and the repair outcomes of DSBs in the male germline can be observed in the progeny (Figure 1A). Repair by SSA recreates a functional copy of *dsRed* and the resulting flies are fluorescent red even in the absence of the endonuclease. Repair by NHEJ creates small insertions or deletions at the I-SceI site and the resulting flies are dark even in the presence of endonuclease. These two classes were scored from populations either with or without endonuclease in order to distinguish them from the uncut reporter carrying flies, which are mosaic red and often undistinguishable from germline DsRed flies [30]. Repair by HR using the sister chromatid (HR-s) recreates the reporter, which can be re-cut until a terminal outcome is reached. HR repair using the homologous chromosome (HR-h) can occur when a Rr3 variant, EJ1, which contains a mutated I-SceI site is present on the homologous chromosome. Repair by HR-h results in dark flies even when the endonuclease is present and can be distinguished from NHEJ by PCR specific to the mutated I-SceI site.

We measured two additional parameters of DSB repair. First, we monitored the extent of gene conversion to the left of the break by determining if the repaired chromosome included a 16 bp deletion 156 bp away from the DSB that was specific to the parental EJ1 chromosome. We designate repaired chromosomes containing the 16 bp deletion as being repaired by long-tract HR-h. Long-tract HR-h repair likely represents repair in which there is increased gene conversion due to increased DNA synthesis rates following strand invasion or due to increased resection prior to DNA synthesis. Alternatively, changes in the repair of the heteroduplex DNA could also influence the balance of repair products observed. Second, large deletions to the left of the DSB result in progeny with white eyes due to the loss of the *white* gene carried on the Rr3 transgene. We performed the Rr3 assay with and without the EJ1 chromosome in order to measure the repair products when repair by HR-h is available and unavailable. All hypothesis testing was done using *P*-values derived from permutation tests described in [31], which account for potential clustering of outcomes in single males due to multiple sperm produced from a single repair event.

In cross 2, in which the EJ1 chromosome was present and the HR-h pathway was available, we saw highly significant decreases in HR-h repair in *brca2* mutants (*P* <1e^−8^) (Figure 1B). Short-tract HR-h was decreased 64-fold from wild-type values and long-tract HR-h was decreased 9-fold. These values likely underestimate the requirement for *brca2* in HR-h. Due to the female sterility of *brca2,* repair pathway use was measured in the genetically-null offspring of females heterozygous for *brca2* and low levels of Brca2 protein may still have been present at the onset of the zygotic transcription when the endonuclease was initially expressed. We also observed a 5-fold increase in flanking deletions in *brca2* mutants. We did not see a significant increase in deletions in cross 1 in which HR-h was not available (Figure 1C). Therefore, the deletions most likely resulted from aberrant HR-h repair, as has been previously suggested for *spnA* and *okra* mutants [32]. We did not observe a requirement for *brca2* in SSA. In fact, there was a significant increase in SSA in *brca2* mutants in both cross 1 and 2 (*P* =1.4e^−7^ and *P* <1.0e^−8^ respectively, Figure 1B,C).

We observed significant changes in SSA and NHEJ in cross 1 when HR-h was unavailable (*P* =1.4 e^−7^ and *P* =4.3 e^−4^) (Figure 1C). In cross 1, the use of SSA increased to 111% of wild-type levels and NHEJ decreased to 74% percent of wild-type levels in *brca2* mutants. It is possible to interpret this result as a partial requirement for *brca2* in NHEJ. However in light of similar results with other HR-h defective mutants [32], our similar results for *okra* mutants (Figure 1C), and the fact that we did not see a decrease in NHEJ in cross 2 (*P* =0.50, Figure 1B), we favor the explanation that changes in NHEJ and SSA levels in cross 1 were due to defects in HR-s that were indirectly measured by changes in NHEJ and SSA. Even though HR-s was not measured, HR-s repair is likely initiated in *brca2* mutants. If DSBs that abort HR-s in *brca2* and *okra* mutants in cross 1 are shuttled specifically into the SSA pathway like the aborted HR-h DSBs in *brca2* and *okra* mutants in cross 2, then the effect would be to increase SSA relative to NHEJ in cross 1. Similar arguments have been proposed for the effects seen in *spnA* mutants [32].

In conclusion we found that *brca2* is required for HR-h and that the loss of HR-h is associated with an increase in the use of SSA repair. In addition, the rate of deletion formation near DSBs increases in *brca2* mutants. These changes in DSB repair represent a shift towards more mutagenic repair of DSBs in *brca2* mutants that are similar to changes seen in *spnA/rad51* and *okra/rad54* mutants ([32] and Figure 1). Therefore we do not find evidence for a Rad51-independent role for Drosophila Brca2 in somatic DNA repair.

### Failure to Repair Meiotic DSBs in *brca2* Mutants Leads to Partial Activation of the Meiotic Recombination Checkpoint

**Figure 2:**
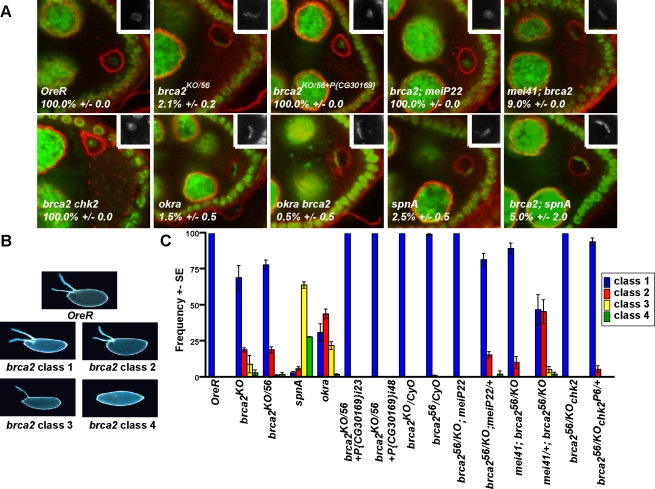
Persistent Double-Stranded Breaks in *brca2* Mutants Activate the Meiotic Recombination Checkpoint (A) Egg chambers stained for DNA (green) and membranes (red). Percentage shown is the average number of wild-type-like, spherical karyosomes from two independent experiments with a total of 175–200 nuclei counted. Insert shows the region containing the oocyte nucleus. (B) Eggshells laid by wild-type and *brca2* ^KO/56^ mutant females. (C) Frequency of the different classes of eggshells laid by females fed yeast for 3–4 days. Class 1 eggs have two separate dorsal appendages and appear wild-type with respect to dorsal patterning. Class 2 eggs have 2 dorsal appendages that are fused at the base. Class 3 eggs have a single dorsal appendage. Class 4 eggs have no dorsal appendages and represent the complete loss of dorsal fate. Error bars represent the standard error between 3 independent experiments. Total number of eggs counted per genotype was between 500 and 3,000 eggs. *P(CG30169}i23* and *P(CG30169}i48* are genomic rescue constructs inserted at two different chromosomal locations.

Mutations in genes required for repair of meiotic DSBs, such as *rad51/spnA,* cause female sterility due to activation of a meiotic checkpoint that affects dorsal-ventral patterning of the eggshell and embryo [1,14]. The DNA endonuclease Mei-W68 catalyzes DSB formation in the germarium of the Drosophila ovary as the initiating step in meiotic recombination [33]. If the DSBs persist, such as in DNA repair mutants, a checkpoint is activated that results in the inefficient translation of Gurken, the ligand for Epidermal Growth Factor receptor responsible for specifying dorsal fates in the Drosophila embryo and eggshell [1,34]. Activation of the meiotic recombination checkpoint results in the ventralization of eggs laid by females mutant for meiotic DSB repair genes. A second characteristic phenotype of checkpoint activation is the failure to properly form the karyosome of the oocyte nucleus. These defects can be suppressed by mutations in the kinases required for checkpoint transduction indicating that they are due to checkpoint activity [1]. Egg chambers from *brca*2 mutant females had a highly penetrant karyosome defect (Figure 2A). In addition, *brca2* mutant females laid weakly ventralized eggs that did not hatch (Figure 2B and 2C). The eggshell patterning and karyosome formation defects can be rescued by a single copy of a genomic rescue construct (Figure 2A and 2C).

Transduction of the meiotic recombination checkpoint signal requires Hus1, a member of the Rad9-Hus1-Rad1 checkpoint complex (9-1-1), and the checkpoint kinases Mei-41/ATR and Chk2 [1,35,36]. Mutations in either *mei-P22*, a gene required for DSB formation [37], or the checkpoint kinase *chk2* suppress the eggshell and karyosome defects of *brca2* mutants (Figure 2A and 2C), clearly demonstrating that the ovarian phenotypes observed are due to persistent DSBs that activate the meiotic checkpoint. We observed little or no suppression of either the eggshell or the karyosome defects in *mei41^D3^*; *brca2^KO/56^* (Figure 2C) or *mei41^29D^*; *brca2^KO/56^* (data not shown) double mutants. This may be due to a possible redundancy between the two upstream checkpoint kinases Mei-41/ATR and ATM. Currently it is not possible to test for a role of *atm* in the meiotic recombination checkpoint as even the hypomorphic, viable *atm* mutants available have eggshell patterning and karyosome defects ([38] and our unpublished results). Additional explanations for the lack of *mei-41* suppression of the *brca2* ovarian phenotype are presented in the Discussion. Nevertheless, suppression by *chk2* mutations indicate that the ovarian phenotypes of *brca2* mutants result from checkpoint activation.

### Brca2 Is Also Required for Efficient Activation of the Meiotic Recombination Checkpoint

**Figure 3:**
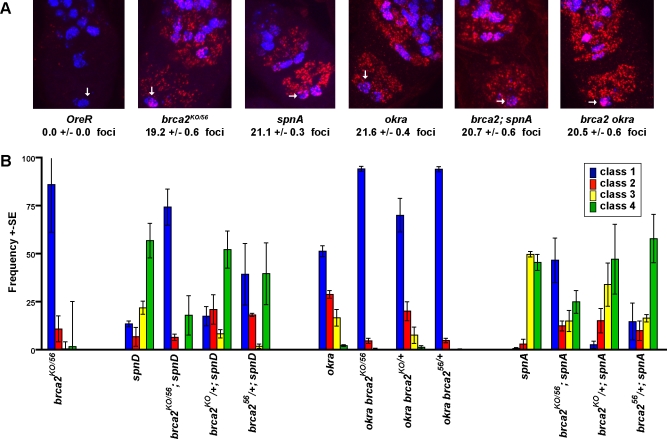
*brca2* Mutants Suppress the Patterning Defects of DNA Repair Mutants That Activate the Meiotic Recombination Checkpoint (A) γ-H2AV(red) foci in the germarium, with the oocyte in region 3 marked by the arrow. Pro-oocytes are marked by C(3)G staining (blue). Images represent a projection of stacks taken through whole germaria. Number of γ-H2AV foci in region 3 is the average number of foci +/− standard error from a total of 14, 14, 11, 7, 10, and 10 region 3 oocytes counted for OreR, *brca2, spnA, okra, brca2; spnA,* and *brca2 okra*, respectively. (B) Frequency of the different classes of eggshells laid by females fed yeast for 5–7 days. Classes are as described in Figure 2. Note the increase in the wild-type like eggshells (blue) in the double mutants. Error bars represent the standard error between 3 independent experiments. Total number of eggs counted per genotype were: 1149, 73, 1687, 456, 645, 1663, 922, 1199, 341, 339, 455, 268, and 196, respectively.

The *brca2* eggshell patterning defect is a considerably weaker defect than that of any of the meiotic DNA repair mutants known to activate the checkpoint, *spnA/rad51*, *spnB/xrcc3*, *spnC/hel308*, *spnD/rad51C*, or *okra*/*rad54* [14,39–41]. We examined the levels of γ-H2AV, a marker for DSBs, in order to determine if the number of DSBs was reduced in *brca2* mutants relative to other meiotic DSB repair mutants, or “spindle class” genes. In wild-type flies γ-H2AV foci are seen in pro-oocyte nuclei in regions 2a and occasionally 2b and are absent from region 3 [42]. An average of 14–15 γ-H2AV foci are present in a pro-oocyte nucleus in region 2a before declining in region 2b. Meiotic DNA repair mutants show a delay in γ-H2AV foci formation, but reach a peak number of 20–24 γ-H2AV foci in region 3 [42]. *brca2* mutants had 19.2 γ-H2AV foci as compared to 21.1 foci in *spnA* mutants in region 3 (Figure 3A). Therefore it is unlikely that the weak phenotype seen in *brca2* mutants is due to decreased accumulation of persistent DSBs.

Surprisingly, when we made double mutants with *brca2* and any of the other meiotic DNA repair mutants, *brca2* strongly suppressed the eggshell phenotype. For example, *spnD* mutant females lay 79.3% strongly ventralized eggs (class3 and class 4 eggs) and 13.8% wild-type-like eggs (class1). *brca2; spnD* mutant females lay 18.7% strongly ventralized eggs and 79.3% wild-type like eggs. Similar results were seen with *okra* and *spnA* mutants (Figure 3B). In order to determine if the suppression of the eggshell phenotype was due to increased DSB repair in the double mutants, we examined the number of γ-H2AV foci present in the double mutants. *spnA*, *okra*, and the double mutants with *brca2* all had similar levels of γ-H2AV foci in region 3 of the germarium (Figure 3A). Therefore it is unlikely that increased levels of repair occur in the double mutants, and we propose that Brca2 has a second role in meiosis in transducing the meiotic recombination checkpoint signal.

### *brca2* and *hus1* Are Not Required for Checkpoints That Respond to Irradiation Damage

The DNA damage checkpoints that respond to multiple types of damage and result in diverse damage responses converge upon a common set of related transducer kinases and mediator proteins [43]. However, each checkpoint has specific genetic requirements for signal transduction. Therefore, we thought it possible to gain a better understanding of where in the meiotic recombination checkpoint pathway Brca2 acts by examining whether *brca2* was required for other Drosophila checkpoints. We used the well-established assays in the imaginal discs and brain of the Drosophila larvae to monitor the checkpoints that respond to irradiation in animals null for *brca2*. Irradiated imaginal discs exhibit a G2 cell cycle arrest within 1 hour of irradiation and induce apoptosis within 4 hours post-irradiation [44,45]. The induction of apoptosis requires *chk2* and *p53*, but not *mei-41* or *grp/chk1* [46]. Cell cycle arrest after high dose IR (4,000 Rads) requires *mei-41*, to lesser extents *chk2* and *grp*, but not *atm* [38,44,46]. Cell cycle arrest after low dose IR (500 Rads) requires *mei-41* as well as *atm* [47]. A *mei-41*-dependent decrease in replication, or an intra-S phase checkpoint, is seen within 2 hours post-irradiation in the larval brain lobes [48].

**Figure 4:**
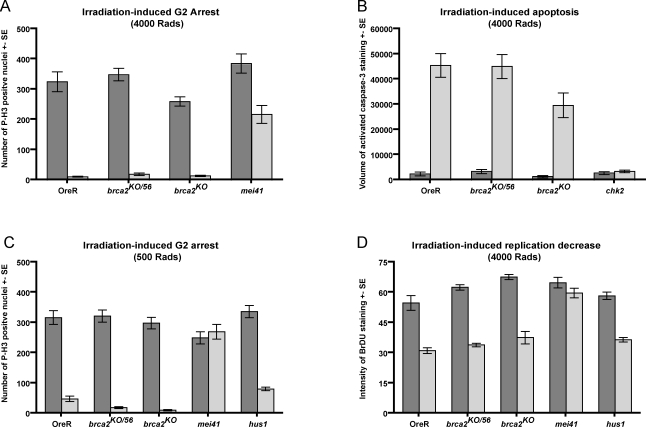
*brca2* Is Not Required for the Checkpoints That Respond to Irradiation Damage Climbing third instar larvae were irradiated and then dissected prior to fixation. Error bars represent the standard error from a total of 6–10 samples from 2–3 separate experiments. Representative images can be found in Figure S1. (A) Larvae were fixed 1 hour after a 4,000 Rad IR exposure and stained for the mitotic marker phospho-histone H3. n = 6. (B) Larvae were fixed 4 hours after a 4,000 Rad IR exposure and stained for activated Caspase-3. n = 6. (C) Larvae were fixed 1 hour after a 500 Rad IR exposure and stained for the mitotic marker phospho-histone H3. n = 11. (D) Larvae were inverted 1.5 hours after a 4,000 Rad IR exposure and incubated in Schneider's media with BrdU for 30 minutes. n = 7–10.

We did not see any requirement for *brca2* in the checkpoints that respond to irradiation (Figures 4 and S1). After a 4,000 Rads IR exposure, *brca2* mutants demonstrated efficient G2 arrest (Figure 4A) and induction of apoptosis (Figure 4B). Cell cycle arrest after a 500 Rad IR exposure was also not compromised in *brca2* mutants (Figure 4C). We did not see any difference between *brca2* mutants and wild-type larvae in the reduction of 5-bromo-2-deoxyuridine (BrdU) staining after a 4,000 Rad exposure (Figure 4D). However, unlike the cell cycle and apoptosis assays in which the difference before and after irradiation is dramatic, the intra-S checkpoint effect is a reduction in replication levels by slightly less than half (Figure 4D). Therefore we do not expect to be able to detect partial requirements for genes involved in the intra-S phase checkpoint. Collectively, the irradiation checkpoints we tested required *atm*, *mei-41/atr*, *chk1*, and *chk2* and did not require *brca2*, demonstrating that the activation of the core checkpoint transducers in response to irradiation in Drosophila does not require *brca2*.

In addition to testing for a requirement for *brca2* in the irradiation checkpoints we also examined *hus1* mutants. Hus1 is a member of the 9-1-1 complex required for activation of the Drosophila meiotic recombination checkpoint and has been shown to be involved in S-phase checkpoints in mammals [36,49]. Similar to *brca2*, *hus1* is not required for the cell cycle arrest or apoptotic induction after high dose IR in Drosophila [36]. In this present study we found that *hus1* is also not required for the G2/M checkpoint after low dose IR or the intra-S checkpoint (Figure 4C and 4D). While we observed a slight increase in the number of mitotic nuclei in *hus1* mutants relative to wild-type after low dose irradiation, we still observe a 4-fold decrease in the number of mitotic cells after IR as compared to unirradiated discs, indicating that the G2/M checkpoint is largely intact in *hus1* mutants after low dose IR.

### Brca2 Interacts with Rad9

**Figure 5:**
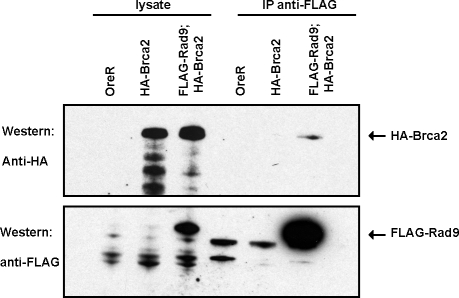
Brca2 Co-Immunoprecipitates with Rad9, a Member of the 9-1-1 Checkpoint Complex Western blots from FLAG-Rad9 immunoprecipitations probed for FLAG and HA epitopes. Approximately 1/20 of the lysate prior to immunoprecipitation and the entire immunoprecipitated protein fraction was loaded. Ovaries from females carrying single copies of *P(UAS-Brca2-HA}* and/or *P(UAS-FLAG- Rad9}* and the germline driver *nanos*-Gal4-VP16 were harvested for the immunoprecipitation.

As *brca2* and *hus1* mutants are both required for efficient activation of the meiotic recombination checkpoint but are not required for the irradiation checkpoints, we wondered if Brca2 acted in concert with the 9-1-1 complex to enforce checkpoint control. We found that epitope-tagged Rad9, a member of the 9-1-1 complex, and Brca2 expressed in the germline cells of the Drosophila ovary co-immunnoprecipitated (Figure 5). Immunoprecipitations for FLAG-tagged Rad9 pulled down HA-tagged Brca2. We roughly estimate from 3 independent experiments that between 1/40 (data not shown) to ∼ 1/400 (Figure 5) of the total HA-Brca2 protein was co-immunnoprecipitated by FLAG-Rad9. It could be that only a subset of the Rad9 present in all regions of the Drosophila ovary binds Brca2. Alternatively, the interaction between Brca2 and Rad9 may be stage-specific, only occurring in the region of the germarium in which recombination intermediates are present and in which a transient checkpoint response may occur. The physical interaction of Brca2 and Rad9 and the similarities in genetic requirements for *hus1* and *brca2* in Drosophila and mammalian checkpoints strongly suggest a common role for Brca2 and the 9-1-1 complex in checkpoint function.

## Discussion

### Drosophila Brca2 as a Model for Human BRCA2

Our results demonstrate that the predicted gene *CG31069* is a functional *BRCA2* homolog required for meiotic and mitotic homologous recombination. We have made null mutations in Drosophila *brca2* in two distinct genetic backgrounds and rescued the ovarian defects by genomic rescue. Unlike in mammals in which null mutations are early embryonic lethal [50], Drosophila *brca2* null mutants are homozygous viable, possibly because of the long period of maternal gene expression during Drosophila embryonic development. It is still uncertain whether Drosophila Brca2 contains a cryptic DNA binding domain similar to mammalian BRCA2 or if this function is encoded in a tightly regulated interacting protein. Further biochemical studies will be necessary to resolve this question, but it is clear from our functional analysis of the role of Brca2 in DNA repair, as well as a recently reported physical interaction with SpnA/Rad51[51], that Drosophila *brca2* represents a functional homolog of the human breast cancer susceptibility gene. Clearly, due to the viability of Drosophila *brca2* null mutants and the power of Drosophila genetics, Drosophila offer a promising new opportunity for uncovering novel roles for BRCA2 during development. In this work we presented a thorough characterization of the role of Brca2 in DSB repair and we uncovered a novel function for Brca2 in the meiotic recombination checkpoint.

### Drosophila Brca2 and Repair

Using the Rr3 assay [30] we showed that in *brca2* mutants, DSB repair is shifted towards repair by potentially mutagenic repair pathways. Repair by homologous recombination is dramatically decreased in *brca2* mutants, and repair by single-strand annealing predominates. Repair by SSA always results in the loss of the sequences between annealed repeats. SSA repair is restricted to DSBs flanked by repetitive elements, though due to the highly repetitive nature of higher eukaryotic chromosomes, SSA repair can represent a significant source of mutagenesis in higher eukaryotes. Our results contrast with studies in C. elegans, in which indirect in vivo experiments and in vitro annealing experiments[16,25] have lead to the suggestion that the C. elegans BRCA2 homolog is required for SSA repair. Our results are more similar to the effects seen in mammalian cell culture experiments with hypomorphic *BRCA2* mutations, in which decreases in HR repair correlated with increases in SSA repair [26–29]. In addition, Brough et al. (2008) also have recently reported a similar inverse relationship between SSA and HR-h in a Drosophila *brca2* mutant using a simplified DSB repair assay [51].

In the Rr3 assay when one pathway is compromised, the sum of the relative pathways usage in these mutants still equals near 100%, even though the percentages are calculated from different populations and are not forced to equal 100% ([32] and our own work). This observation plus the fact that different effects on repair pathway balance have been observed among mutants with decreases in the same pathway suggest that regulated compensation can occur. For example, mutations in *mus101* and *mei-41* both result in a decrease in SSA, but the former are compensated by increases in NHEJ and HR-h, while the latter is compensated by NHEJ only [32]. In cross 2, we saw a significant increase in the use of the SSA (*P* <1e^−8^), but no significant difference in the relative level of NHEJ (*P* = 0.50) in *brca2* mutants (Figure 1B), indicating compensation by the SSA pathway in *brca2* mutants.

Compensation of decreases in HR-h by the SSA pathway seems to be a common response to deficiencies in genes required for HR-h. In mammalian cell culture studies, Stark et al. (2004) found a similar inverse relationship between SSA and HR in *Brca2* and *Rad51* mutants, while *Brca1* mutants had decreases in both SSA and HR [29]. Using the Rr3 assay, Johnson-Schlitz et al*.* (2007) found that the significant decreases in HR-h in Drosophila *dmBlm*, *top3α*, and *spnA* mutants were compensated entirely through increases in SSA, while in *okra* mutants compensation occurred through significant increases in both SSA and NHEJ. In our experiments we observed compensation in *okra* mutants entirely through SSA, and the exact cause of the discrepancy is unclear. Possibly the discrepancy lies in the use of different endonuclease sources or in different combinations of *okra* alleles used (*okra* ^AA/RU^ versus *okra* ^RU/WS^), though mutagen sensitivity studies have suggested that the AA and WS alleles are of similar strength [40]. Regardless, it is clear that *brca2* mutants are compensated by SSA, similar to most mutants deficient for HR-h in Drosophila and other organisms.

It is also notable that short-tract HR-h is more strongly affected in *brca2* mutants than long-tract HR-h. Short-tract HR-h was decreased 64-fold relative to wild-type values and long-tract HR-h was decreased 9-fold (Figure 1B). The residual HR-h repair probably reflects repair that occurred early when low levels of maternal Brca2 were still present and wild-type levels of Brca2 may be required to restrict the extent of gene conversion during HR-h repair. Gene conversion tract length during HR-h repair has important implications in maintaining genomic integrity as the potential for loss of heterozygosity increases with increasing tract length. Mutations in *okra* led to a similar shift towards long-tract HR-h (Figure 1B), and homozygous and heterozygous mutations in *spnA* have also been observed to alter the balance of HR-h in towards long-tract HR-h [32]. These results suggest that reductions in levels of the enzymes required for strand invasion can result in increased rates of loss of heterozygosity during HR-h repair. Increased long-tract HR-h repair relative to short-tract HR-h repair in *brca2* mutants may represent increased rate of DNA synthesis, increased resection prior to strand invasion, increased stability of recombination intermediates, unequal repair of the heteroduplex DNA, or a combination of these processes. In mammalian cells, an increase in the extent of gene conversion has been observed in both *Rad51^K133R^* and *Xrcc3*, a *Rad51* paralog, mutants; though these observed increases are thought to have arisen from different mechanisms due to differences seen in gene conversion tract continuity [29,52]. Models explaining the increase in the inclusion of the 16 bp deletion in Drosophila *brca2* and *spnA* mutants would first need to determine whether conversion tracts in these mutants are continuous or discontinuous by using a more complicated reporter design.

### Drosophila Brca2, the 9-1-1 Complex, and Checkpoints

We found a novel requirement for *brca2* in transduction of the meiotic recombination checkpoint signal. Initially the meiotic phenotypes of *brca2* mutants were surprisingly different from the spindle class mutants previously studied. First, even with the exacerbation of the ventralization defect by growth at 25 °C, the eggshell ventralization phenotype of *brca2* mutants was significantly weaker in spite of similar levels of persistent DSBs (Figure 2). Second, the kinetics of the eggshell phenotype were opposite to the kinetics of classical spindle mutants. In *spnA,B,C,D* and *okra* mutants the phenotype is weak during the initial days in which the females are fed yeast, but after 5–7 days on yeast spindle mutants lay predominately severely ventralized eggs. In *brca2* mutants, ventralized eggs were only reliably laid during the first 1–4 days on yeast (data not shown). Given our results, it is now clear that the requirement for *brca2* in efficient transduction of the checkpoint signal masks the strong eggshell ventralization phenotype that is normally suggestive of a role in meiotic DSB repair. As an increasing number of proteins with dual roles in DNA repair and checkpoint function are being identified, it will be interesting to see if there are additional dual function proteins functioning in Drosophila meiosis. While the classical meiotic repair mutants have strong oogenesis phenotypes, weak or absent eggshell patterning defects may not preclude a role in meiotic DNA repair if coupled to a role in checkpoint transduction.

We found that the ovarian phenotypes of *brca2* mutants were suppressed by mutations in *chk2*, but were not suppressed by *mei-41* mutations. This finding is in contrast to the other spindle class mutants which are suppressed by both *mei-41* and *chk2* mutations [1,14,39]. Our model is that in females with an intact checkpoint response, the checkpoint activation is dependent upon *mei-41* and *chk2*. However, because Brca2 acts in a similar step in the checkpoint pathway as Mei-41, no additional suppression is observed in the *mei-41; brca2* double mutant. Since both the eggshell and karyosome defects of *brca2* mutants can be suppressed by *chk2* it is probable that the residual checkpoint activation in *brca2* mutants is due to activation of Chk2 by the upstream checkpoint kinase Atm. It is not currently possible to test the involvement of Atm in the checkpoint as even viable, hypomorphic *atm* single mutants have eggshell and karyosome defects. There is however evidence that Mei41-independent checkpoints exist in Drosophila meiosis. Upregulation of transposable elements in the Drosophila germline, as seen in *cutoff* mutants, results in eggshell patterning defects that can be suppressed by *chk2* but not by *mei-41* mutations [53]. The checkpoint activated in *cutoff* mutants is, however, distinct in at least some aspects from the checkpoint activated in *brca2* mutants. Unlike in *brca2* mutants, the checkpoint activated in *cutoff* mutants results in a loss of germline cells as well as eggshell patterning defects, and these defects cannot be suppressed by mutations that prevent DSB formation.

According to our model, Mei41-independent checkpoint activation in *brca2* mutants is strong enough, with respect to either signal strength or signal duration, to result in a karyosome defect but not strong enough to result in strong eggshell patterning defects. Clearly Mei-41 is responsible for the bulk of checkpoint activation in classical spindle mutants but it remains possible that Atm may play a supporting role. It is notable that the classical spindle phenotypes are typically scored after 5–7 days on yeast at ambient temperature and that at these conditions the *brca2* single mutant phenotype was very weak (data not shown). Therefore it is possible that under the conditions used in this study *mei-41* mutations may also not completely suppress the classical spindle mutations. In interpreting the role of *brca2* in the transduction of the meiotic recombination signal we have had to focus on the eggshell phenotype as *brca2* single mutants have karyosome defects. This phenotype is similar to that seen in *hus1* mutant females. *hus1* mutants are able to suppress the eggshell defects of spindle mutants but do not suppress the karyosome defects of the spindle mutants as *hus1* single mutants have karyosome defects [36]. Though it has not been tested in this study, it seems possible that, similar to *brca2* mutants, the *hus1* mutant karyosome defect is a result of persistent DSBs and partial activation of the checkpoint.

We also found that Brca2 co-immunoprecipitated with Rad9. The 9-1-1 complex forms a heterotrimeric ring that is loaded onto resected single-stranded DNA flanking a DSB following replication stress [54,55]. The 9-1-1 complex associates with DSBs independent of ATR, and mediates Chk1 phosphorylation by ATR through interaction with TopBP1[56–59]. The 9-1-1 complex may or may not use a similar mechanism to activate Chk2 during the meiotic recombination checkpoint. Although the precise functional relevance of the Rad9-Brca2 interaction remains to be explored, our co-immunoprecipitation results further suggest that the role of Brca2 in checkpoint control is upstream of Chk1/Chk2.

Research in mammals and our work in Drosophila has shown that the 9-1-1 complex and BRCA2 are specifically required for the checkpoints thought to be activated in response to large stretches of single-stranded DNA. In Drosophila we have shown that they are required for the meiotic recombination checkpoint, but not the irradiation checkpoints. In mammals, BRCA2 and the 9-1-1 complex are required for several S-phase checkpoints [3,10,49]. Now that a physical connection between Rad9 and Brca2 has been observed, it will be interesting to determine the degree of functional overlap between Brca2 and the 9-1-1 complex in the DNA damage responses. In light of the absence of a predicted DNA binding domain in Drosophila *brca2* it is tempting to predict that the Drosophila 9-1-1 complex and Brca2 may have a common role in both repair and checkpoints. While the functional overlap is complete for the checkpoints examined to date, it is clear from mutagen sensitivity assays that the functional overlap between 9-1-1 and Brca2 in DNA repair is not absolute. *hus1* mutants are severely sensitive to MMS, but not to IR, while *brca2* mutants are moderately sensitive to MMS and severely sensitive to IR ([36] and this study).

In conclusion we have found that the Drosophila homolog of *BRCA2* is required for mitotic and meiotic homologous recombination and in the absence of *brca2* error-prone repair predominates. *brca2,* similar to the 9-1-1 complex with which it physically interacts, has a second requirement during meiosis in the activation of the meiotic recombination checkpoint but is not required for checkpoints that respond to irradiation induced damage, indicating a specialized role for *brca2* in checkpoint control.

## Methods

### Fly strains and transgenes.

Flies were maintained at 25 °C. Additional information about culturing conditions can be found in Text S1. *brca2* ^56E^ was isolated by screening for imprecise excisions after mobilization of *P(SUPor-P}CG30169^KG03961^* which was inserted in the 5'UTR of *CG30169*. *brca2* ^56E^ removes the majority of the coding sequence of *CG30169* and most of the 5'UTR of *CG4612*; the sequence junction is GTGGTGGCGGCT/ AACTTGCCGGCAA. *brca2* ^KO^ was created by ends-out homologous recombination using the targeting vector pW25 [21]. Flanking homologous sequences were amplified, inserted into the BsiWI/AscI and Acc65I/SphI sites of pW25, and predicted open reading frames were verified by sequencing. The sequence junction of the deleted region is: TTGTTTTGGAAATGC/ AAGGTGCCCACTTAC. The structure of the resulting knock-out was verified by PCR and Southern blot analysis (data not shown). To make *P(CG30169}* a 5.8 Kb XhoI-NotI fragment containing *CG30169* was isolated from the BAC clone BACR11c07 (BACPAC Resource Center) and ligated into pCasper4. To make *P(UAS-Brca2-HA}* the region containing the coding sequence of Brca2-PA was PCR-amplified using primers that created a 5' NotI site and a 3' XbaI site. Three copies of HA followed by BglII and KpnI sites were placed downstream of the XbaI site. The resulting NotI-KpnI fragment was cloned into pUASp. To make the *P(UAS-FLAG- Rad9}*, the coding sequence of Rad9-PA was amplified using primers to create a 5' KpnI site and a 3' XbaI site. The FLAG peptide sequence was added at the 5' end following the KpnI site. The resulting KpnI -XbaI fragment was cloned into pUASp. *nanos*-Gal4-VP16 driven Brca2-HA expression can rescue the eggshell patterning defects of *brca2* mutants (data not shown). P-element-mediated germ-line transformation was carried out according to standard protocols [60]. The following previously-described allele combinations were used: *mei-41^D3^* (except where noted), *chk2^P6^*, *spnA^57^*/*spnA^093^*, *okra^AA^*/*okra^RU^*, *spnD^150^*, *atm^red31^*, *hus1^37^*. Details about the alleles can be found at http://flybase.bio.indiana.edu. The *P(Rr3}48C*, *P(Rr3EJ1}48C*, and *TM3 Sb P(UIE}72C* transgenic flies were obtained from Bill Engels [30]. The following flies were made by recombination: *P(Rr3}48C wg^SP-1^ L brca2^56E^*, *P(Rr3}48C wg^Sp-1^ L okra^RU^*, *P(Rr3EJ1}48C brca2^K0^*, and *P(Rr3EJ1}48C okra^AA^*.

### Antibody stainings.

Ovaries were fixed for 20 minutes in 1:3 4% paraformaldehyde/ PBS:heptane and other tissues were fixed in 4% paraformaldehyde/PBS and blocked in 3% BSA/PBS and further processed according to standard protocols. The following modifications were made: the γ-H2AV stainings were blocked in 10% goat serum and P-H3(Ser28) stainings were fixed for 1 hour. Primary antibodies were incubated overnight at 4 °C in PBS + 0.3% Triton (PBST) + 1% BSA or 10% goat serum at the following concentrations: 1:500 rabbit α-γ-H2AV, 1:1000 rat α-Encore, 1:500 mouse α-C(3)G clone 1A8-1G2, 1:500 rabbit α-Cleaved Caspase-3 (Cell Signaling), 1:100 mouse α-BrdU clone 3D4 (BD Biosciences), 1:500 α-P-H3(Ser28) (Upstate). 1:1000 Secondary antibodies (Molecular Probes), 1:10,000 Hoechst (Molecular Probes), or 1μg/ml Wheat-germ Agglutinin-488 (Molecular Probes) were incubated 2 hours in PBST.

### Repair reporter 3 assay.

Details on the Rr3 assay can be found in [30–32]. Briefly, individual males bearing the paternally inherited endonuclease *TM3 Sb P(UIE}72C* and the reporter constructs, *P(Rr3}48C* and *P(Rr3EJ1}48C*, were backcrossed to *w* ^1118^ virgins. All matings occurred within two weeks of eclosion to minimize age-related effects. *L wg* ^SP−1^ progeny were scored for eye color, presence of the endonuclease, and DsRed florescence. In cross 1, in which the EJ1 chromosome was not present, SSA repair was scored as the percent of DsRed positive non-endonuclease-bearing (endo**^−^**) progeny/ all endo**^−^** progeny. NHEJ was scored as the percent of DsRed-negative, endonuclease-bearing (endo**^+^**) progeny/ all endo**^+^** progeny. In cross 2 in which both Rr3 and EJ1 were present, SSA repair was again scored as DsRed-positive endo**^−^**/ all endo**^−^**. The DsRed-negative endo**^+^** flies represented repair by either HR-h or NHEJ and were distinguished by PCR. We used primers to specifically amplify the region containing the mutated I-SceI site on the EJ1 chromosome [30]; presence of a band indicated repair by HR-h. We also used control primers in the same PCR reaction to exclude rare unsuccessful reactions. PCR products were run on a 3% MetaPhor agarose gel (Lonza) to simultaneously distinguish between repair by short and long tract HR-h. Ten Dark endo^+^ progeny per male or as many as were available were used for PCR. Deletions were scored as percent of white-eyed males among total Rr3 progeny; the means and standard errors were multiplied by 2 to allow comparisons with previously reported values. The deletion class was not isolated prior to DsRed and PCR scoring and therefore also represents a small portion of the NHEJ class. Permutation tests were used to compute two-tailed *P*-values as described in [31].

### Mutagen sensitivities.

Heterozygous parents were mated and eggs were collected for 24 hours at room temperature in vials for chemical mutagenesis or apple juice plates for irradiation. 48 hours later the larvae were treated with 250 μl of 0.025% or 0.08% methyl methanesulfonate (Sigma) or irradiated with 500 or 1,250 Rads in a Faxitron X-ray cabinet. Control flies were treated with 250 μl water or not irradiated. After eclosion the percent of homozygous mutant flies was determined, and the sensitivity was expressed as the percent of mutant flies/ total progeny in the treated vial as compared to the relative percent of mutant flies in untreated control vials.

### Checkpoint assays.

Climbing third instar larva were irradiated at 500 or 4,000 Rads in a Faxitron X-ray cabinet. For analysis of cell cycle progression and apoptosis, larva were inverted in PBS and fixed after 1 and 4 hours respectively. For analysis of replication, larva were inverted in Schneider's media 1.5 hours after irradiation. Larvae were incubated with Schneider's media + 10 μM BrdU (Sigma) for 30 minutes, washed 2 times 3 minutes and fixed in 4% paraformaldehyde/PBS for 20 minutes. Larvae were washed and then incubated in 2N HCL for 30 minutes, 100 mM borax for 2 minutes, washed and then blocked and stained as described above. Confocal stacks of 0.5 μm intervals were analyzed using Volocity 3DM software (Improvision) for cell cycle and apoptosis assays and ImageJ for the replication assay. To quantitate the BrdU stainings, the mean grayscale value of three evenly-spaced regions of 1,400 square pixels in the wide nuclei-dense bands of the brain lobe were averaged for each brain lobe.

### Co-immunoprecipitation and Western blots.

Epitope-tagged Rad9 and Brca2 were expressed using the germline Gal4 driver *nanos*-VP16 [61]. 10 ovary pairs were ground in 150 μl IP buffer (50 mM Tris pH 8, 150mM NaCl, 1mM CaCl2, 0.1% Tween-20) supplemented with complete mini protease inhibitor EDTA-free (Roche) and type I and II phosphatase inhibitors (Sigma). Cleared lysate was incubated at 4 °C for 2 hours with mouse αFLAG M2 antibody (Sigma) bound to 20 μL Protein A/G PLUS Agarose slurry (Santa Cruz Biotechnology) after a 30 minute preincubation with an equal volume of Protein A/G beads without antibody. Fusion proteins were detected using mouse αFLAG-M2-HRP conjugate (Sigma) at 1:2000 and rat αHA-HRP conjugate (Roche) at 1:3000 for Western analysis.

## Supporting Information

Figure S1 *brca2* Is Not Required for the Checkpoints That Respond to Irradiation Damage in the Drosophila Larval Wing Discs and Brain Lobes Climbing third instar larvae were irradiated and then dissected prior to fixation. Representative images are projections of 0.5 micron interval stacks of control tissues (A-R) or tissues that were irradiated prior to fixation (A'-R'). (A,E,I,M,A',E',I',M') Larvae were fixed 1 hour after a 4,000 Rad IR exposure and stained for the mitotic marker P-H3. (B,F,J,N B',F',J',N′) Larvae were fixed 4 hours after a 4,000 Rad IR exposure and stained for activated Caspase-3. (C,G,K,O,Q,C',G',K',O',Q'). Larvae were fixed 1 hour after a 500 Rad IR exposure and stained for the mitotic marker P-H3. (D,H,L,P,R,D',H',L',P',R') Larvae were inverted 1.5 hours after a 4,000 Rad IR exposure and incubated in Schneider's media with BrdU for 30 minutes. Genotypes tested were: (A–D') OreR, (E–H') *brca2* ^KO/56^, (I–L') *brca2* ^KO^, (M,M',O-P”) *mei41*, (N,N′) *chk2*, and (Q–R') *hus1*.
(3.4 MB TIF)

Text S1 Supplemental Methods (21 KB DOC)

## Abbreviations

9-1-1: Rad9-Rad1-Hus1 complex
bp: base pair
BrdU: 5-bromo-2-deoxyuridine
DSB: double-stranded break
HR: homologous recombination
HR-h: homologous recombination using the homologous chromosome
HR-s: homologous recombination using the sister chromatid
IR: irradiation
MMS: methyl methanesulfonate
NHEJ: non-homologous end joining
Rr3: Repair reporter 3
SSA: single-strand annealing
