# Supplementary material for: Drosophila brca2 Is Required for Mitotic and Meiotic DNA Repair and Efficient Activation of the Meiotic Recombination Checkpoint
Source: PLoS Genet. 2008 Feb 8;4(2):e31. doi: 10.1371/journal.pgen.0040031 (PMC2233675; doi:10.1371/journal.pgen.0040031)
Supplement: Supplementary file 1 [file pgen.0040031.sd001.doc]

Text S1. Supplemental Methods. Flies were maintained at 25°C in uncrowded conditions. Previous experiments using spindle mutants involved flies raised at ambient temperature. Consequently, the percent ventralized eggshells reported in other papers should not be compared directly to experiments presented here, as this phenotype depends on both the temperature and days on yeast. Importantly, for this reason the relevant controls are reported with each experiment. We find that culturing at higher temperatures (25°C versus 20-22°C) increases both the severity of the phenotype of all spindle mutant eggshell phenotypes and the reproducibility of weaker eggshell phenotypes, such as *brca2* mutants. The eggshell phenotypes of the classical spindle mutants (*spnA,B,D* and *okra*) are often weak during the first 1-3 days on yeast and strengthen and stabilize by days 5-7 on yeast, when the phenotype is typically scored. The karyosome defect is not sensitive to the feeding regime or the temperature. We find that it is important to score both phenotypes in order to assess checkpoint activation. *brca2* mutants behave atypically from classical spindle mutants in that the eggshell phenotype is the strongest and most reproducible during the first 1-4 days on yeast (data not shown). Therefore we first characterized the *brca2* mutant eggshell phenotype during days 3-4 (Figure 2); but scored on days 5-6 when examining double mutants with other spindle mutants in order to examine the spindle phenotype when it was the most stable (Figure 3). Further, the γ-H2AV stainings were done after 4 days on yeast.
